# Antitumor effects of cecropin B-LHRH’ on drug-resistant ovarian and endometrial cancer cells

**DOI:** 10.1186/s12885-016-2287-0

**Published:** 2016-03-28

**Authors:** Xiaoyong Li, Bo Shen, Qi Chen, Xiaohui Zhang, Yiqing Ye, Fengmei Wang, Xinmei Zhang

**Affiliations:** Department of Gynecology, Women’s Hospital, School of Medicine, Zhejiang University, Hangzhou, China; Institute of Radiation Medicine, Fudan University, Shanghai, China; Central Laboratory, Women’s Hospital, School of Medicine, Zhejiang University, Hangzhou, China; Department of Women Health Care, Women’s Hospital, School of Medicine, Zhejiang University, Hangzhou, China; Pharmacy Division, Women’s Hospital, School of Medicine, Zhejiang University, Hangzhou, China

**Keywords:** Luteinizing hormone releasing-hormone receptor, Cecropin B peptide, Ovarian cancer, Endometrial cancer

## Abstract

**Background:**

Luteinizing hormone-releasing hormone receptor (LHRHr) represents a promising therapeutic target for treating sex hormone-dependent tumors. We coupled cecropin B, an antimicrobial peptide, to LHRH’, a form of LHRH modified at carboxyl-terminal residues 4–10, which binds to LHRHr without interfering with luteinizing hormone (LH) and follicle-stimulating hormone (FSH) secretion. This study aimed to assess the antitumor effects of cecropin B-LHRH’ (CB-LHRH’) in drug-resistant ovarian and endometrial cancers.

**Methods:**

To evaluate the antitumor effects of CB-LHRH’, three drug resistant ovarian cancer cell lines (SKOV-3, ES-2, NIH:OVCAR-3) and an endometrial cancer cell line (HEC-1A) were treated with CB-LHRH’. Cell morphology changes were assessed using inverted and electron microscopes. In addition, cell growth and cell cytotoxicity were measured by MTT assay and LDH release, respectively. In addition, hemolysis was measured. Furthermore, radioligand receptor binding, hypersensitization and minimal inhibitory concentrations (against *Staphylococcus aureus, Klebsiella pneumoniae, Escherichia coli, Enterobacter cloacae, Pseudomonas aeruginosa, and Acinetobacter baumannii*) were determined. Finally, the impact on tumor growth *in BALB/c-nu* mice was assessed in an ES-2 xenograft model.

**Results:**

CB-LHRH’ bound LHRHr with high-affinity (dissociation constant, Kd = 0.252 ± 0.061nM). Interestingly, CB-LHRH’ significantly inhibited the cell viability of SKOV-3, ES-2, NIH:OVCAR-3 and HEC-1A, but not that of normal eukaryotic cells. CB-LHRH’ was active against bacteria at micromolar concentrations, and caused no hypersensitivity in guinea pigs. Furthermore, CB-LHRH’ inhibited tumor growth with a 23.8 and 20.4 % reduction in tumor weight at 50 and 25 mg/kg.d, respectively.

**Conclusions:**

CB-LHRH’ is a candidate for targeted chemotherapy against ovarian and endometrial cancers.

## Background

Sex hormone-dependent tumors, including ovarian, endometrial, breast and prostate carcinomas, are the most common reproductive system tumors. Ovarian cancer is often detected at a late stage [1–3]; despite cytoreductive surgery and paclitaxel/platinum-based chemotherapy it frequently recurs, resulting in poor prognosis [4–6]. Although endometrial carcinoma presents at an early stage and responds well to surgery [7, 8], frequent recurrence also results in poor prognosis [8, 9]. Chemotherapy of sex hormone-dependent cancers is limited by the intrinsic or acquired drug resistance of tumor cells as well as the toxicity to normal cells of chemotherapeutic agents [5, 10, 11], whose side effect profiles limit the chemotherapeutic dosing [12, 13].

Targeted cytotoxic agents enable selective treatment of primary tumors and their metastases, reducing side effects and improving efficacy [2, 14–17]. Luteinizing hormone-releasing hormone receptor (LHRHr) may represent a useful target in sex hormone-dependent tumors as it is expressed in human breast (52 %), ovarian (80 %), endometrial (80 %), and prostate (86 %) carcinomas [18]. Potential drawbacks of targeted chemotherapy using natural LHRH as a binding partner include side effects resulting from interference with pituitary secretion of LH and FSH [19]. Previous LHRHr targeted cytotoxic therapies include cytotoxins, such as doxorubicin, which have non-specific cytotoxicity. Other LHRHr targeted therapies used chemical approaches linking the cytotoxin to LHRH; however, such conjugates are readily hydrolyzed in the blood stream, releasing cytotoxic radicals before reaching their therapeutic targets, therefore inducing non-specific cytotoxicity [19]. Refined and improved strategies for LHRHr chemotherapy are necessary if these approaches are to be useful.

LHRH is a decapeptide that binds to receptors on pituitary gonadotropes, stimulating biosynthesis and secretion of FSH and LH, which regulate gonadal steroidogenesis and gametogenesis in both sexes [20]. The carboxyl-terminal residues 4–10 of LHRH are involved in receptor binding, while amino-terminal residues 1–3 activate the receptors [21]. As targeted chemotherapy using natural LHRH as a carrier may interfere with LH and FSH secretion, we constructed a modified peptide, LHRH’, in which amino-terminal residues 1–3 are not included. Thus, LHRH’ is expected to target LHRHr positive carcinoma cells, while not interfering with LH and FSH secretion.

Cecropin B is an antimicrobial peptide (AMP) first characterized in 1980 [22, 23]; since then, thousands of similar molecules have been isolated from a wide range of organisms [24, 25]. AMPs are short peptides possessing net cationic charges, selective toxicity, rapid cytotoxic effects, broad antimicrobial spectra, and no documented resistance [24–26]. Some AMPs, including cecropins, are highly potent against cancer cells but not normal mammalian cells [27–31], making them attractive for the treatment of some cancers.

In this study, we developed a novel LHRHr cytotoxin by linking Cecropin B to the modified LHRH’ receptor ligand, Cecropin B-LHRH’ (CB-LHRH’) [32]. We hypothesized that the novel CB-LHRH’ may bind to LHRH receptors and deliver an effective broad-spectrum toxin specifically to tumor cells, without affecting healthy cells or readily inducing resistance. Therefore, we aimed in this study to assess the antitumor effects of CB-LHRH’ in the treatment of drug-resistant ovarian and endometrial cancers, using BALB/c-nu mice (a common model for cancer studies) harboring ES-2 xenografts.

## Methods

The study was approved by the ethical committee of Women’s Hospital, School of Medicine, Zhejiang University, Hangzhou, China. Informed consent was obtained from all individual participants included in the study.

### Peptides and cytotoxic agents

CB-LHRH’ recombinant polypeptide (KWKVFKKIEKMGRNIRNGIVKAGPAIAVLGEAKALSYGLRPG) (Shanghai Sangon Biological Engineering Technology & Services Corporation, Shanghai, China) was synthesized by solid phase synthesis to a purity of 96 % as evaluated by high-performance liquid chromatography. It was identified by mass spectrographic analysis (MW 4,566.57D), dissolved in phosphate-buffered saline (PBS), and diluted to the desired concentration before use.

### Drug-resistant tumor cell lines

Four drug-resistant cancer cell lines, including three ovarian cancer cell lines (SKOV-3, ES-2, NIH:OVCAR-3) and one endometrial cancer cell line (HEC-1A) (Cell Bank of Shanghai Biological Institute, Shanghai, China), were cultured in RPMI 1640 with 10 % fetal bovine serum, 1,000 IU/mL penicillin, and 100 mg/mL streptomycin at 37 °C and 5 % CO_2_. Cell cultures and subculture procedures were performed according to the recommendations of the ATCC Global Bioresource Center. ES-2, HEC-1A, and NIH:OVCAR-3 cells express LHRHr, while the SKOV-3 does not express LHRHr [33–35].

### Clonogenic assay

A clonogenic assay was carried out as previously described [36]. 300 cells were seeded into 6-well dishes in 2 mL of medium. After overnight incubation, CB-LHRH’ was added at a final concentration of 12.5 μM and cells were further cultured for 10 d. Cells were then stained with crystal violet (0.5 % w/v). Colonies containing a minimum of 50 cells were counted using a dissection microscope. Each treatment was performed in triplicate and each experiment repeated three times.

### Cytotoxicity assay

Lactate dehydrogenase (LDH) release was quantified using LDH Cytotoxicity Detection Kit (Roche Applied Science, Mannheim, Germany) according to the manufacturer’s instructions. Briefly, 100 μL of exponentially growing tumor cells (6 × 10^4^ cells/mL) were seeded into 96-well plates, cultured overnight, and washed with RPMI 1640. Then, several CB-LHRH’ amounts (6.25, 12.5, 25, 50, and 100 μM) were completed to 100 μL with RPMI and added to cells, followed by 4 h incubation. LDH release was assessed by measuring absorbance at 490 and 630 nm, respectively, on a microplate reader (Bio-Tek Instruments Inc., Vermont, USA). Cytotoxicity was determined by the following formula: Cytotoxicity (%) = (exp. value - low control)/(high control - low control) x 100. All conditions were carried out in triplicate, and the experiments repeated three times.

### Proliferation assay

The anti-proliferative activity of CB-LHRH’ was assessed by the 3-(4,5-dimethylthiazol-2-yl)-2,5-diphenyl-tetrazolium bromide (MTT) assay [37]. Briefly, 100 μL of exponentially growing tumor cells (6 × 10^4^ cells/mL) were seeded in 96-well plates and cultured overnight. B-LHRH’ was then added to each well at final concentrations of 6.25, 12.5, 25, 50, and 100 μM. After another 24 or 48 h, 10 μL of fresh MTT (Sigma–Aldrich, St. Louis, USA) at 5 mg/mL in Hank’s salt solution was added for 4 h. After careful removal of the medium, 100 μL dimethylsulfoxide (Sigma–Aldrich, St. Louis, USA) was added to wells followed by absorbance reading on a microplate reader (Bio-Tek Instruments Inc, Vermont, USA) at 490 and 630 nm, respectively. Cell viability was determined as follows: Cell viability (%) = (absorbance of treated wells - absorbance of blank control)/(absorbance of negative control - absorbance of blank control) x 100. The inhibition rate was derived as 1-Cell viability. Each experiment was performed in triplicate and repeated three times.

Cell morphology was monitored using inverted and electron microscopes after 1 h exposure to CB-LHRH’ as previously described [38].

### Electron microscopy

For transmission electron microscopy ES-2 cells grown on sterile slides were fixed with 2.5 % glutaraldehyde in pH 7.4 phosphate buffer for 1 h at 4 °C, followed by post-fixation in 1 % osmium tetroxide 1 h at 4 °C and dehydrated in an alcohol gradient (50 to 100 %). The attached cells were then dried by Lyophilization and coated with Au before examination with transmission electron microscope (S-4800 SEM, Hitachi, Ltd. Tokyo, Japan).

For scanning electron microscopy ES-2 cells were grown and fixed under similar conditions, without slides. After post-fixation cells were infiltrated with Epon resin and sectioned with Leica UC6 ultramicrotome (Leica Microsystems Inc., LKB-II, Wetzlar, Germany). Sections of about 100 nm were post-stained with lead citrate and uranyl acetate. Grids were examined with scanning electron microscope (JEM-200CX TEM, JEOL, Ltd. Tokyo, Japan).

### Cytotoxicity of CB-LHRH’ in normal eukaryotic cells

The ability of CB-LHRH’ to induce cytotoxicity in normal eukaryotic cells was assessed as previously described [39]. With informed consent and institutional review board approval, peripheral blood samples were obtained from healthy human donors. Red blood cells were separated by centrifugation at 1,000 g for 5 min followed by three washes with PBS. Washed red blood cells were resuspended in sterile Alsever’s solution (2.05 % dextrose, 0.8 % sodium citrate, 0.055 % citric acid, and 0.42 % sodium chloride, pH 6.1, hematocrit 5 %) in a plate and treated with CB-LHRH’ at 100, 200, 300, 400, or 500 μM for either 30 min or 4 h at 37 °C with shaking. After centrifugation for 10 min at 600 g, the supernatant was collected for hemolysis measurement at 490 nm (hemoglobin absorbance). Adding equal volumes of water to red blood cells provided 100 % hemolysis (positive control); cell-free Alsever’s solution was used as negative control. Each experiment was performed in triplicate and repeated three times.

### Radioligand receptor binding assays

Under approved animal research protocol standards, binding capacity to LHRHr was assessed in rat pituitary membranes by radioligand-binding assays as previously described [40]. The experiment was approved by the animal care and use committee of Fudan University.

#### Preparation of membrane fractions for primary pituicytes

To obtain primary pituicytes, SD male and female rats (180–240 g) were sacrificed and the pituitary glands dissected and washed twice with 10 mM Tris-HCl buffer, pH 7.4, containing 0.5 mM PMSF, 1.2 mM MgCl_2_, 0.01 mM EDTA-Na_2_. Tissue aliquots were homogenized using an automatic glass Potter homogenizer at 2000 rpm for 3 × 10 s. After nucleus and debris removal by centrifugation at 2000 g and 4 °C for 5 min, membrane preparation aliquots were collected by centrifugation at 2000 g and 4 °C for 20 min. Protein concentrations were determined by the Lowry method using bovine serum albumin (BSA) as a standard, diluted with 10 mM Tris-HCl buffer to 2 mg/mL stock at −70 °C.

#### Radioligand receptor binding assay

CB-LHRH’ peptide was radioactively labeled with Iodine-125 (Perkin Elmer Life and Analytical Sciences, Waltham, USA) using Chloramine-T (Sigma–Aldrich, St. Louis, USA) and purified on Sephadex G15 chromatography (Sigma–Aldrich) using 0.1 mM aqueous acetic acid, containing 2.5 g/L BSA as eluent. The specific activity of ^125^I-CB-LHRH’ was about 0.2 ~ 0.24 mCi/mmol with a purity greater than 95 %. In the binding assay, polypropylene tubes were pretreated overnight at 4 °C with 10 mM Tris-HCl, pH 7.4 containing 1 % BSA. Membrane fraction aliquots (100 μg protein in 50 μL per tube) were incubated with different concentrations of ^125^I-CB-LHRH’ at 37 °C for 1 h in a total volume of 150 μL. To examine the binding specificity, 2 μg gonadorelin (Tash Biotechnology, Shanghai, China) was used as competitor. Reactions were terminated by adding 1 mL of 10 mM Tris-HCl buffer (pH 7.4, 4 °C). The solutions were subsequently filtered onto Whatman filters, washed with 1 mL of 5 % trichloroacetic acid and transferred to a counting tube. The radioactivity was measured by a gamma counter (GC-300, AnHui ustc ZonKia Scientific Instruments co. LTD, Hefei, China). Specific binding was determined by subtracting nonspecific from total binding. The equilibrium dissociation constant (Kd) and maximum binding capacity (Bmax) were derived by the Scatchard method. Kd represents the concentration of drug inducing the half (50 %) biggest effect. Bmax indicates the maximum binding volume combined per mg receptor proteins. Each experiment was performed in triplicate and repeated three times.

### Hypersensitization test

Under approved animal research protocol standards, the hypersensitive reaction against CB-LHRH’ (2 mg/mL, equal to 438 μM) was assessed in guinea pigs. According to the Chinese Pharmacopoeia, six animals with an average weight of 250 g (XiFengYang Special Economic Animal Farm, Huzhou, China) were administered an intraperitoneal injection of 0.5 mL of CB-LHRH’ every other day three times, while three additional animals received PBS physiological saline injections. Of the six animals administered CB-LHRH’, three received an intravenous injection of 1 mL CB-LHRH’ at day 15, and the remaining three at day 22. Allergic reactions, such as piloerection, sneezing, lacrimation, dyspnoea, and hyperspasmia, were then monitored for the next 30 min.

### Antibacterial assay

The antibacterial activity of CB-LHRH’ was evaluated by the agar dilution method [41]. Muller Hinton’s agar medium plates containing serial dilutions of CB-LHRH’ were inoculated with 2 μL of tenfold-diluted 0.5 McF bacteria and incubated for 22 h at 35 °C. The lowest concentration of CB-LHRH’ inhibiting bacterial growth was considered the minimal inhibitory concentration (MIC). Nine clinical strains of the following species were evaluated: *Staphylococcus aureus, Klebsiella pneumoniae, Escherichia coli, Enterobacter cloacae, Pseudomonas aeruginosa, and Acinetobacter baumannii*.

### Xenograft studies

Five- to six-week-old female (15–20 g) athymic nude mice (BALB/c-nu) were obtained from Shanghai SLAC Laboratory Animal Corporation, China. The animals were housed in sterile cages under laminar flow hoods in a temperature controlled room with a 12-hour light/12-hour dark cycle, and fed autoclaved chow and water *ad libitum*. All experiments were carried out in accordance with the guidelines for the welfare ethics of experimental animals [42]. All the animal-related procedures were approved by the Animal Ethical Committee of Zhejiang University.

#### Experimental protocol

Cultured ES-2 ovarian cancer cells were resuspended at 1 × 10^7^/ml and inoculated subcutaneously into the nude mouse armpit. When tumors reached 60 mm^3^, they were extracted, cut into pieces of about 2 mm^3^, and inoculated subcutaneously into the nude mouse armpit. When the tumors reached an average volume of about 100 mm^3^, the mice were randomly divided into five groups (*n* = 10). Groups 1–3 received intra-tumor injection of CB-LHRH’ twice daily at 12.5, 25 or 50 mg/kg^.^d, respectively. Group 4 received 2 mg/kg cisplatin, once every 3 days [43], and group 5 the same volume of PBS, administered twice daily.

Tumor volumes were assessed every 3 d as 0.5 × length × width^2^ (where length is the longest diameter across the tumor and width the corresponding perpendicular diameter as measured by calipers). On day 13, 4 h after the last dose, mice were sacrificed, and tumors excised and weighed; tumor growth inhibition rate was derived as (1-tumor weight treated/tumor weight control) × 100 %.

#### Response criteria

Anti-tumor activity was evaluated as relative growth of tumor volume (T/C, value), the mean relative tumor volume (RTV) for the treatment group divided by the mean RTV for the control group, as follows: T/C (%) = T_RTV_/C_RTV_ X 100 %. RTV = V_t_/V_0_, where V_t_ is the volume on any given day, and V_0_ the volume at treatment start. Agents producing a T/C of >60 % were considered to be inactive, those with a mean T/C of ≤60 % considered to be active.

General toxicity was evaluated based on body weight, measured weekly.

### Statistical analysis

Data are mean ± standard deviation (SD). Normality and homogeneity of variance assumptions were assessed. One way analysis of variance (ANOVA) was used for multiple group comparison, as required. SPSS 16.0 (SPSS, USA) was used for the statistical analysis. *p* < 0.05 was considered statistically significant.

## Results

### CB-LHRH’ inhibited proliferation of cancer cell lines

Three drug-resistant ovarian cancer cell lines (SKOV-3, ES-2, NIH:OVCAR-3) and one drug-resistant endometrial cancer cell line (HEC-1A) were incubated with 12.5 μM CB-LHRH’. After 10 d there were almost no colonies after CB-LHRH’ treatment, while control wells contained approximately 30–50 colonies, indicating that 12.5 μM CB-LHRH’ significantly inhibited the growth of drug-resistant cancer cells.

**Fig. 1:**
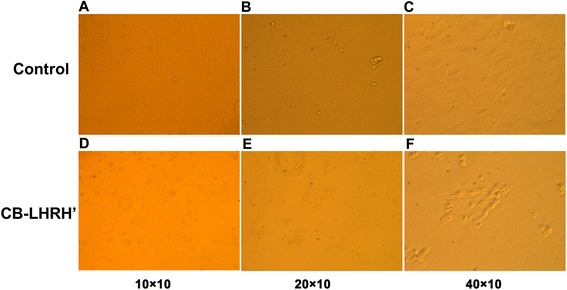
Cecropin B-LHRH’ induces changes in ES-2 morphology. Untreated cells (**a**–**c**, 10, 20, and 40×, respectively), display normal morphology, while cells treated with 25 μM cecropin B-LHRH’ for 24 h (**d**–**f**, 10, 20, and 40×, respectively) showed reduced number, and aggregated, detached from the plate, inflated, released cytoplasm, and disintegrated into granules. Morphological changes observed in ES-2 cell line

Damage to all four cell lines was observed within 24 h of exposure to 12.5 μM CB-LHRH’ by light microscopy (data not shown). And the effect of CB-LHRH’ on ES-2 cell morphology was shown in Fig. 1. Typical cell damage included aggregation, detachment from the plate, inflation, cytoplasm leak, disintegration into granules, and decreased cell number (Fig. 1).

**Fig. 2:**
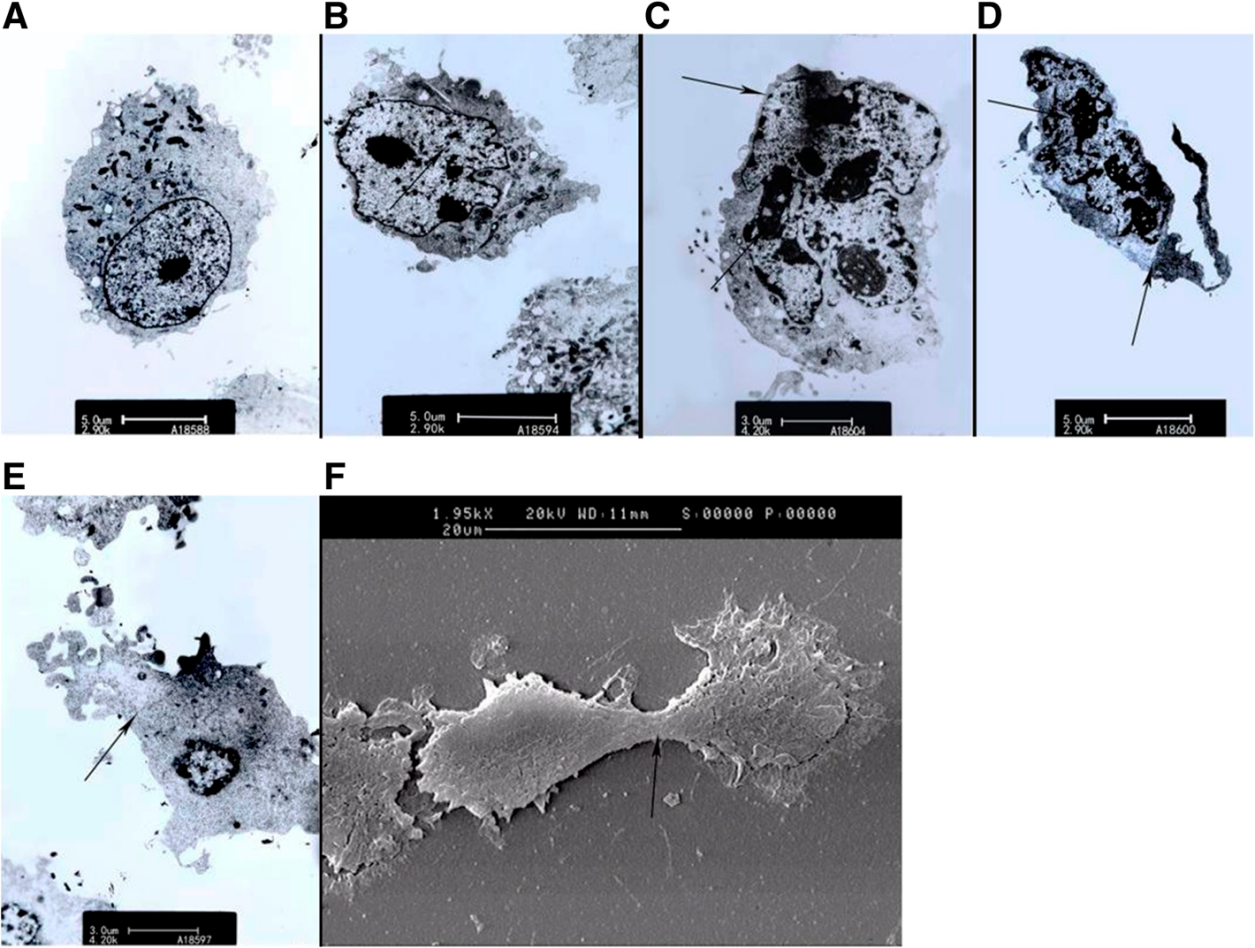
Cecropin B-LHRH’ induces ultrastructural changes in ES-2 cells. Transmission electron microscope images (**a**–**e**), and scanning electron microscope images (**f**) of a normal ES-2 cell (**a**); ES-2 cells incubated with 25 μM cecropin B-LHRH’ for 1 h (**b**–**f**). Figures **b**–**d** illustrate cell membrane blebbing, the disappearance or reduction of microvilli, cell shrinkage, increased cellular granularity, and the formation and separation of apoptotic bodies. **e** and **f** show cytoplasm leak. Morphological changes observed in ES-2 cell line

Electron microscopy revealed cell membrane blebbing, the disappearance or reduction of microvilli, cell shrinkage, increased cellular granularity, the formation and separation of apoptotic bodies, and cytoplasm leak in response to 25 μM CB-LHRH’ in ES-2 cell (Fig. 2).

**Fig. 3:**
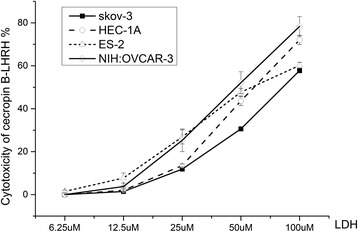
Cytotoxicity of cecropin B-LHRH’ against the four cancer cell lines. Cytotoxicity was detected in SKOV-3, ES-2, NIH:OVCAR-3, and HEC-1A cells incubated overnight with CB-LHRH’ by LDH Cytotoxicity Detection Kit. Concentration-dependent cytotoxicity was observed in all four cell lines

All four cell lines incubated for 4 h with CB-LHRH’ exhibited concentration-dependent cytotoxicity (*P* < 0.001) as shown in Fig. 3. And there were no differences among the four cell lines (Fig. 3). In contrast, CB-LHRH’ caused no hemolysis of red blood cells from healthy human donors (data not shown).

**Fig. 4:**
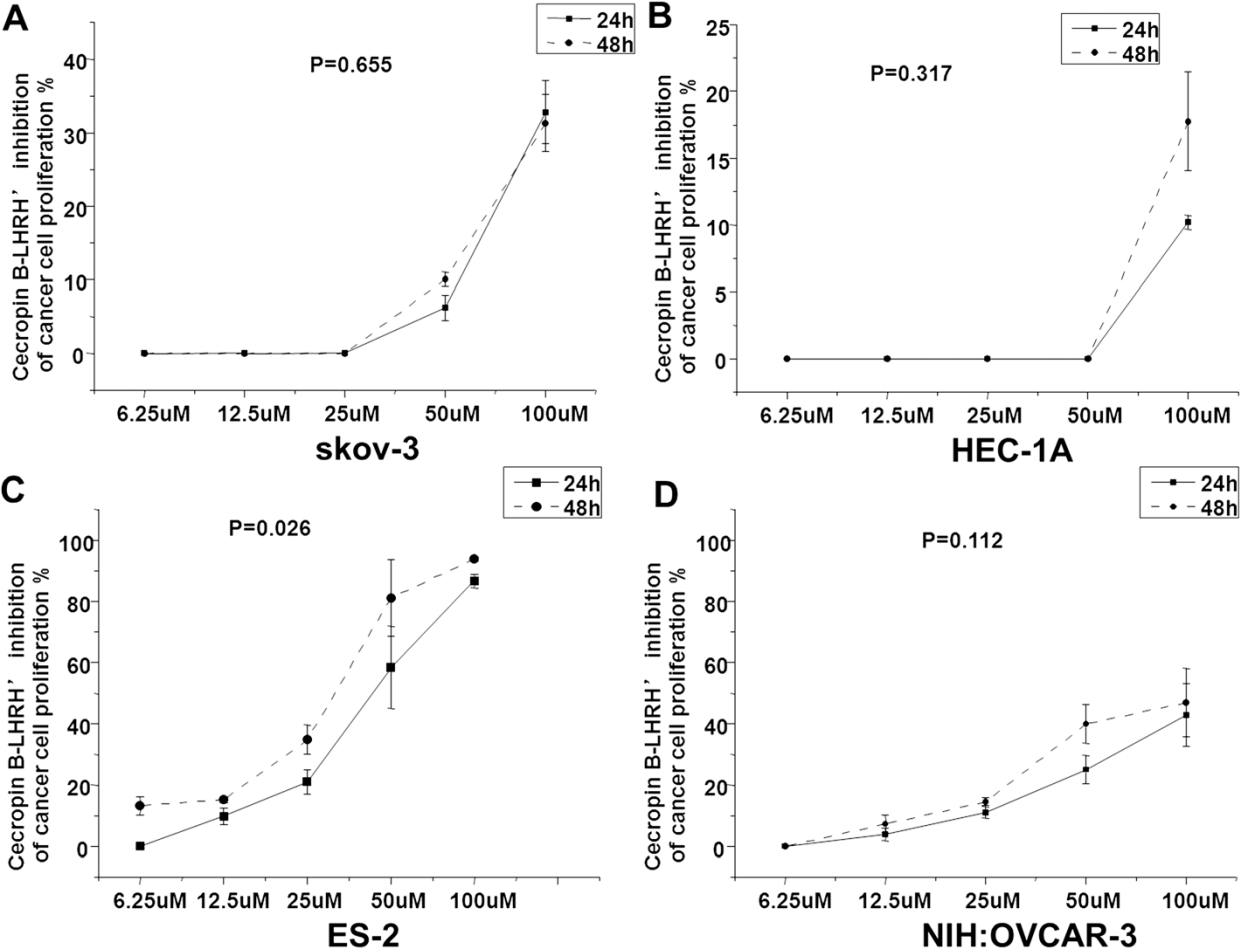
Cecropin B-LHRH’ inhibition of cancer cell proliferation. Proliferation inhibition was detected in (**a**) SKOV-3, (**b**) HEC-1A, (**c**) ES-2 and (**d**) NIH:OVCAR-3 cells incubated with CB-LHRH’ at the dose of 6.25 μM, 12.5 μM,25 μM,50 μM, and 100 μM for 24 h or 48 h by the MTT assay. Proliferation was inhibited in a time- and concentration-dependent manner in ES-2 cell line (*p* = 0.026), and in a concentration-dependent manner in other three cell lines (*p* < 0.001 vs 6.25 μM). Data are presented as mean ± SD, *n* = 3

Proliferation of all four drug-resistant cancer cell lines was inhibited by CB-LHRH’ in a concentration-dependent manner (*P* < 0.001) (Fig. 4a–d). And the greatest inhibition was obtained for ES-2 cells, and CB-LHRH’ inhibited cell proliferation in ES-2 cell in a time-dependent manner (*P* < 0.05) (Fig. 4c). Inhibition of SKOV-3 and HEC-1A cell proliferation was weaker at the dose of lower than 25 μM and 50 μM, respectively (Fig. 4a and 4b). And inhibition of NIH:OVCAR-3 cell proliferation was up to 40 % even at the high dose of 100 μM (Fig. 4d).

### CB-LHRH’ is a high-affinity ligand for LHRHr

**Fig. 5:**
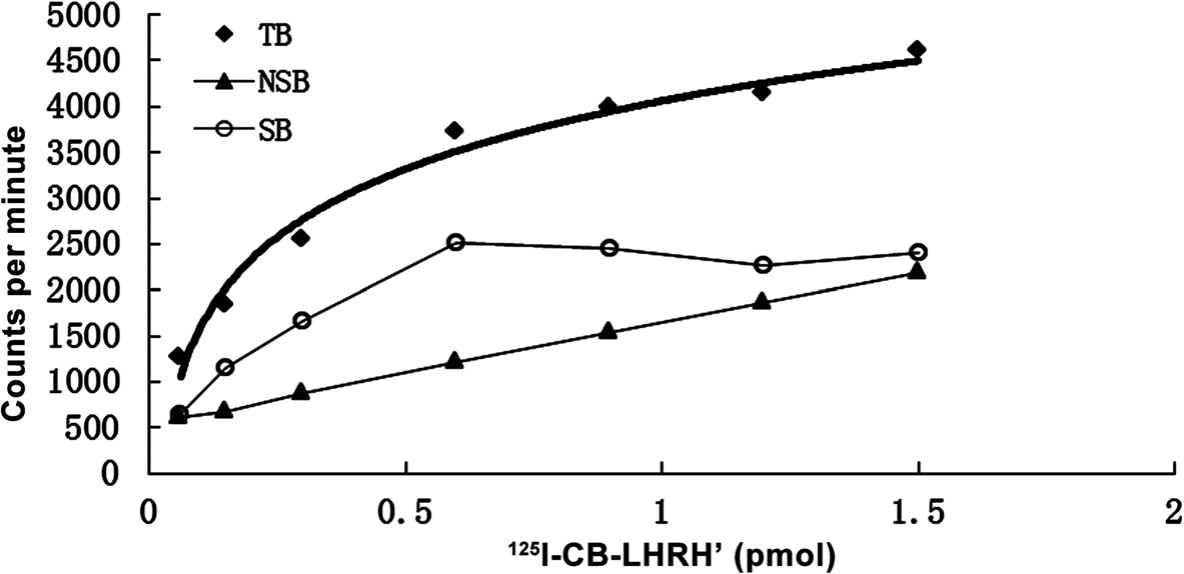
Saturation curve and Scatchard plot of LHRHr-specific ^125^I-cecropin B-LHRH’ binding. The binding affinity of CB-LHRH’ to the LHRH receptor was assessed in rat pituitary membranes by radioligand-binding assays. TB, total binding; SB, specific binding; NSB, non-specific binding. Each experiment was performed in triplicate and repeated three times

The binding affinity of CB-LHRH’ to the LHRH receptor was assessed in rat pituitary membranes by radioligand-binding assays. Kd and Bmax values were 0.2526 ± 0.061 nM and 110.4 ± 17.68 fmol/mg protein, respectively (Fig. 5), indicating that ^125^I-CB-LHRH’ is a high-affinity ligand for LHRHr.

### CB-LHRH’ does not induce hypersensitivity

The potential of CB-LHRH’ to induce a hypersensitive reaction in mammals was assessed in the guinea pig model. Guinea pigs administered 0.5 mL 2 mg/ml CB-LHRH’ intraperitoneally every other day three times (*n* = 6) survived the 15 days of treatment and their weights did not differ significantly from animals administered PBS (*n* = 3). Afterwards, three animals were administrated an additional 1 mL of 2 mg/ml CB-LHRH’. No anaphylaxis occurred in any of the six guinea pigs treated with CB-LHRH’ (data not shown).

### CB-LHRH’ inhibits Gram-negative bacteria

The antibacterial activity of CB-LHRH’ was evaluated by the agar dilution method. CB-LHRH’ killed or inhibited the growth of all tested gram-negative bacteria at micromolar concentrations. The minimal inhibitory concentrations (MIC) were 1.93–7.72 μM, 3.86–7.72 μM, 3.86–7.72 μM, 3.86–7.72 μM, ≥15.44 μM, and >15.44 μM for *Acinetobacter baumannii, Escherichia coli, Enterobacter cloacae*, *Klebsiella pneumoniae, Pseudomonas aeruginosa*, and *Staphylococcus aureus*, respectively (Table 1)*.*

**Table 1:** The MIC of the Cecropin B-LHRH’ against the six kinds of clinically isolated bacteria

Bacteria	MIC (μM)
*Acinetobacter baumannii*	1.93–7.72
*Escherichia coli*	3.86–7.72
*Enterobacter cloacae*	3.86–7.72
*Klebsiella pneumoniae*	3.86–7.72
*Pseudomonas aeruginosa*	> = 15.44
*Staphylococcus aureus*	>15.44

### CB-LHRH’ inhibits ES-2 Xenograft growth in vivo

Nude mice were implanted with ES-2 and administered intra-tumoral injection of CB-LHRH’ twice daily at 0, 12.5, 25 or 50 mg/kg^.^d, or 2 mg/kg cisplatin, once every 3 days (*n* = 10); thirteen days later 9, 8, 9, 7 and 8 surviving mice were obtained, respectively (Tables 2 and 3).

**Table 2:** Therapeutic effect of Cecropin B-LHRH’ on the volume of ES-2 tumors xenografted into nude mice (Mean ± SD)

Group	Dose (mg/kg.d)	Tumor Volume (mm^3^)		RTV	T/C (%)
		d 1	d 13		
CB-LHRH’	50	308.91 + 110.19	965.92 + 212.31**	3.13	50
	25	364.18 + 112.16	1334.78 + 179.25**	3.67	59
	12.5	298.23 + 107.54	1416.71 + 353.71**	4.75	76
Cisplatin	0.67	343.91 + 123.06	913.16 + 142.64**	2.66	42
PBS		326.56 + 85.00	2042.62 + 239.07	6.5	–

In comparison to the PBS control, ** *P* < 0.01

**Table 3:** Therapeutic effects of Cecropin B-LHRH’ on the weight of ES-2 tumors xenografted into nude mice (Mean ± SD)

Group	Tumor Weight (g)	Tumor growth inhibition rate (%)
50 mg/kg^.^d CB-LHRH’	1.31 ± 0.31*	23.8
25 mg/kg^.^d CB-LHRH’	1.37 ± 0.15*	20.4
12.5 mg/kg^.^d CB-LHRH’	1.40 ± 0.81	18.4
Cisplatin	1.09 ± 0.25 **	36.4
PBS	1.72 ± 0.24	-

In comparison to the PBS control, *P < 0.05; **P < 0.01

Each experiment was performed in triplicate and repeated three times

However, administration of CB-LHRH’ at 50 and 25 mg/kg significantly inhibited the growth of ES-2 Xenografts by day 13, resulting in tumor sizes of 50 % and 59 % the control values (*P* < 0.01), and 23.8 % and 20.4 % decrease in tumor weights (*P* < 0.05), respectively; no differences were found in animal weights.

## Discussion

In this study, we developed a novel LHRHr cytotoxin strategy by linking Cecropin B to the modified LHRH’ receptor ligand in order to deliver an effective broad-spectrum toxin specifically to tumor cell targets, without affecting gonadotrophin secretion in healthy cells or inducing resistance.

CB-LHRH’ binds to LHRHr with a low dissociation constant of 0.252 ± 0.061 nM, indicating the high-affinity of the modified peptide, which should have broad-spectrum toxicity against cancer cells expressing LHRHr. Indeed, CB-LHRH’ was cytotoxic to ES-2 and NIH:OVCAR-3 (ovarian cancer cells) and HEC-1A (endometrial cancer cells) which express LHRHr, while displaying the weakest cytotoxicity in the LHRHr negative ovarian cancer cell line SKOV-3. Of note, the four cancer cell lines sensitive to CB-LHRH’ are known to be resistant to several cytotoxic drugs, including diphtheria toxin, doxorubicin, cisplatin, and adriamycin, among others, according to the ATCC Global Bioresource Center (USA). For instance, ES-2 cells were derived from an ovarian clear cell carcinoma characterized by unique clinical features such as high incidence in stage I of the disease, relatively strong resistance to conventional platinum or taxane-based chemotherapies, and poor prognoses [44–46]. The overt cytotoxicity demonstrated for CB-LHRH’ against several multidrug-resistant cancer cell-lines, particularly ES-2 cells, indicates that the new peptide might help in the treatment of some cancers resistant to currently available chemotherapeutics; indeed, CB-LHRH’ is a promising candidate for the treatment of LHRHr dependent cancers.

The mechanisms underlying the antimicrobial and anticancer effects of cecropin include plasma membrane disruption via micellization, or pore formation by peptide-lipid interactions [47, 48]. Cationic peptides target characteristic, cell-surface, anionic lipids by electrostatic attraction that are ubiquitous and exclusive to micro-organisms, followed by membrane permeation and disruption. This simple electrostatic discrimination provides selective toxicity, as well as a broad spectrum of antimicrobial activity. Unspecific molecular recognition makes the development of resistance difficult. Eukaryotic cell membranes have important amounts of sterols, and the membrane-stabilizing cholesterol protects normal eukaryotic cells from attacks by therapeutic peptides [47, 48]. Changes in lipid compositions of cancer cell membranes may be a reason why these peptides show specific toxicity to cancer cells [30]. In this study, loss of cytoplasm and disintegration of cancer cells, with no hemolytic activity in normal red blood cells, supports similar anticancer mechanisms of plasma membrane disruption.

CB-LHRH’ also induced no hypersensitive reactions in guinea pigs, suggesting that CB-LHRH’ may not cause severe allergy, a common problem of peptide drugs. This benign profile distinguishes the newly developed polypeptide from other chemotherapeutics, as the safety of chemotherapeutics is one of the most important features of contemporary drug treatment. However, these results need to be replicated in human subjects in order to rule out potential relevant inter-species differences. In addition, the anticancer effects of CB-LHRH’ was assessed after intra-tumoral administration, which does not fully recapitulate the targeting of tumors in the peritoneal space.

CB-LHRH’ also inhibited the growth of clinically isolated bacteria at micromolar concentrations, indicating a potent antibacterial effect. The antibacterial and anticancer functions of CB-LHRH’ suggest that the carboxyl-terminal extension of CB doesn’t alter its antibacterial properties. In addition to inhibiting growth of cancer cells, CB-LHRH’ may also benefit cancer patients with its antibacterial function, since bacterial infections are a major cause of morbidity and mortality in neutropenic patients following chemotherapy for malignancy [49].

## Conclusion

LHRH’ may represent a more promising therapeutic than LHRH, and cecropin B appears to be a potentially valuable component of targeted therapies. The peptide drug CB-LHRH’ described here is a potent candidate for cancer therapy, with broad anti-cancer spectrum and minimal toxicity to normal eukaryotic cells.

## Ethics approval and consent to participate

The study was approved by the ethical committee of Women’s Hospital, School of Medicine, Zhejiang University, Hangzhou, China. Informed consent was obtained from all individual participants included in the study.

## Consent for publication

Not applicable.

## Availability of data and materials

The datasets supporting the conclusions of this article are included within the article.

## Abbreviations

AMP: antimicrobial peptide
Bmax: maximum binding capacity
BSA: bovine serum albumin
FSH: follicle-stimulating hormone
Kd: equilibrium dissociation constant
LDH: lactate dehydrogenase
LH: luteinizing hormone
LHRH: luteinizing hormone-releasing hormone
LHRH’: modified LHRH
LHRHr: luteinizing hormone-releasing hormone receptor
MIC: minimal inhibitory concentration
MTT: 3-(4, 5-dimethylthiazol-2-yl)-2,5-diphenyl-tetrazolium bromide
PBS: phosphate-buffered salt solution
RTV: relative tumor volume
T/C value: RTV of the treatment group/RTV of the control group
